# Receptor for Advanced Glycation End Products (RAGE) and Mechanisms and Therapeutic Opportunities in Diabetes and Cardiovascular Disease: Insights From Human Subjects and Animal Models

**DOI:** 10.3389/fcvm.2020.00037

**Published:** 2020-03-10

**Authors:** Lander Egaña-Gorroño, Raquel López-Díez, Gautham Yepuri, Lisa S. Ramirez, Sergey Reverdatto, Paul F. Gugger, Alexander Shekhtman, Ravichandran Ramasamy, Ann Marie Schmidt

**Affiliations:** ^1^Diabetes Research Program, Division of Endocrinology, Diabetes and Metabolism, Department of Medicine, New York University School of Medicine, New York, NY, United States; ^2^Department of Chemistry, University of Albany, State University of New York, Albany, NY, United States

**Keywords:** diabetes, obesity, cardiovascular disease, peripheral arterial disease, RAGE, DIAPH1

## Abstract

Obesity and diabetes are leading causes of cardiovascular morbidity and mortality. Although extensive strides have been made in the treatments for non-diabetic atherosclerosis and its complications, for patients with diabetes, these therapies provide less benefit for protection from cardiovascular disease (CVD). These considerations spur the concept that diabetes-specific, disease-modifying therapies are essential to identify, especially as the epidemics of obesity and diabetes continue to expand. Hence, as hyperglycemia is a defining feature of diabetes, it is logical to probe the impact of the specific consequences of hyperglycemia on the vessel wall, immune cell perturbation, and endothelial dysfunction—all harbingers to the development of CVD. In this context, high levels of blood glucose stimulate the formation of the irreversible advanced glycation end products, the products of non-enzymatic glycation and oxidation of proteins and lipids. AGEs accumulate in diabetic circulation and tissues and the interaction of AGEs with their chief cellular receptor, receptor for AGE or RAGE, contributes to vascular and immune cell perturbation. The cytoplasmic domain of RAGE lacks endogenous kinase activity; the discovery that this intracellular domain of RAGE binds to the formin, DIAPH1, and that DIAPH1 is essential for RAGE ligand-mediated signal transduction, identifies the specific cellular means by which RAGE functions and highlights a new target for therapeutic interruption of RAGE signaling. In human subjects, prominent signals for RAGE activity include the presence and levels of two forms of soluble RAGE, sRAGE, and endogenous secretory (es) RAGE. Further, genetic studies have revealed single nucleotide polymorphisms (SNPs) of the *AGER* gene (*AGER* is the gene encoding RAGE) and *DIAPH1*, which display associations with CVD. This Review presents current knowledge regarding the roles for RAGE and DIAPH1 in the causes and consequences of diabetes, from obesity to CVD. Studies both from human subjects and animal models are presented to highlight the breadth of evidence linking RAGE and DIAPH1 to the cardiovascular consequences of these metabolic disorders.

## Introduction

We reported the discovery of the receptor for advanced glycation end products (RAGE; gene name is *AGER*) in 1992 on account of this molecule's ability to bind the products of non-enzymatic glycation and oxidation of proteins/lipids, the advanced glycation end products, or AGEs (1). AGEs are not solely biomarkers of a hyperglycemic and pro-inflammatory/pro-oxidative state; rather, they also play mediating roles in the pathogenesis of diabetic complications, in large part through their interactions with RAGE. Various AGEs are also generated in highly heated and processed foods (2). Hence, AGE interaction with RAGE ensues both from endogenously-formed AGE adducts, as well as from dietary AGE sources. RAGE is expressed on multiple types of cells, such as vascular cells, immune cells, neurons, cardiomyocytes, adipocytes, glomerular epithelial cells or podocytes, lung epithelial cells, and a wide range of transformed cells, both in animal models and human subjects (3–5).

The pivotal discovery in the biology of RAGE was the finding that RAGE bound a diverse series of ligands beyond AGEs, such as members of the S100/calgranulin family, high-mobility group box 1 (HMGB1), lysophosphatidic acid (LPA) and oligomeric forms of amyloid beta peptide (Aβ) and islet amyloid polypeptide (IAPP) (6–10). These ligands bind to the extracellular domains of RAGE in a heterogeneous manner; although the extracellular V-type immunoglobulin (Ig) domain binds to many of the ligand families, the binding sites on the V-domain are multiple and spatially distinct. Further, ligands may also bind at the extracellular C1 and C2-type Ig domains, thereby further diversifying the complexity of the RAGE-ligand interactions (11–14).

Soluble forms of the receptor have also been described (15). Identified as “sRAGEs,” these forms of RAGE have been found in plasma, and in other fluid compartments, such as synovial fluid, cerebrospinal fluid, and bronchoalveolar lavage fluid, as examples (15–18). There are two major forms of sRAGE that result from distinct mechanisms. Most of the circulating sRAGE results from cell surface-cleavage of the full-length receptor by species such as matrix metalloproteinases (MMPs) and a disintegrin and metalloprotease domain-containing protein 10 (ADAM10) (19). G-protein coupled receptor (GPCR) activity has also been linked to the production of sRAGE (20). The other form of sRAGE, known as endogenous secretory or esRAGE, represents a less prevalent form of the sRAGE in plasma and is a product of a splice variant of *AGER* (21).

In the absence of endogenous kinase activity, the means by which the RAGE cytoplasmic domain signals and impacts transcriptional programs and cellular functions remained elusive until the discovery that this RAGE intracellular domain binds the formin, Diaphanous1 (DIAPH1), and that this interaction is essential for RAGE signaling in multiple cell types (22). The cytoplasmic domain of RAGE, particularly through its amino acids R366/Q367, binds to the formin homology 1 (FH1) domain of DIAPH1; mutation of these amino acids to alanine residues or knock-down of *Diaph1* results in loss of this binding and loss of RAGE ligand (but not non-RAGE ligand)-mediated signaling in smooth muscle cells (SMCs) and transformed cells, respectively (22, 23). Others, using super-resolution stochastic optical reconstruction microscopy (STORM) and single-particle tracking (SPT), independently confirmed the interaction of the cytoplasmic domain of RAGE with DIAPH1 (24).

*In vivo*, DIAPH1 has been linked to numerous *in vivo* settings in which RAGE ligands and RAGE have been implicated, such as neointimal expansion after vessel injury, hypoxia-mediated damage, myocardial ischemia, diabetes-associated nephropathy, cancer, responses to infection (such as *Listeria monocytogenes*), and immune/inflammatory responses (25–33). Key downstream effectors of DIAPH1 relevant to cellular perturbation include activation of pathways such as the following: RhoGTPases, such as CDC42, RAC1, and RHOA; glycogen synthase kinase3β (GSK3β) and AKT; Rho-associated, coiled-coil-containing protein kinase (ROCK), Serum Response Factors (SRF); and SRF-dependent genes, such as *Egr1, Tagln*, or *c-fos* (25–33).

In the sections to follow, recent findings linking RAGE to both the pathogenesis and complications of diabetes, particularly in the setting of cardiometabolic dysfunction and disease, will be discussed. Recent developments in the generation of a novel class of RAGE/DIAPH1 antagonists will be presented, as well as opportunities for biomarking cardiometabolic disease through the lens of the RAGE signaling pathway in human subjects.

## CVD, Diabetes, and RAGE/DIAPH1

In both types 1 and 2 diabetes (T1D, T2D), CVD remains a leading cause of morbidity and mortality (34–36). When diabetes is combined with MI or stroke, the mortality rate for affected patients is nearly doubled, leading to an estimated reduction in life expectancy of ~12 years (37). Beyond management of lipids and blood pressure and modulation of life style, major gaps in the therapeutic armamentarium in diabetes and CVD still exist, underscoring the critical need for disease-modifying therapies for these disorders. To follow is a review of common manifestations of CVD and the links to the RAGE/DIAPH1 pathway.

### Atherosclerosis

Numerous studies have illustrated that RAGE is expressed in both non-diabetic and diabetic atherosclerotic lesions in human subjects, but that the expression is higher in diabetes and co-localizes with markers of lesional oxidative and inflammatory stress (38, 39). An ever-growing series of published work associates RAGE with atherosclerosis, both in human subjects and in animal models.

#### Studies in Human Subjects

Levels of sRAGEs have been extensively studied in human subjects to test associations of the RAGE pathway to diabetes and CVD. In a study of T1D subjects and healthy control subjects studied at baseline (age 8–18 years) and after 5 years of follow-up, levels of sRAGE and esRAGE declined with aging, in a manner independent of sex, diabetes, or pubertal stage. In the diabetic subject group, the levels of sRAGE and esRAGE were positively associated with carotid intima-media thickness (IMT) and baseline sRAGE was negatively associated with levels of C-reactive protein (CRP) at the follow-up testing (40). The authors concluded that high levels of baseline sRAGE might protect from inflammation 5 years later, but no protection from abnormalities of arterial stiffness or wall thickness was noted (40).

Recent studies have probed if levels of sRAGE in patients with metabolic dysfunction but without diagnosed diabetes provided surrogate markers for incipient atherosclerosis. Levels of esRAGE were examined in non-diabetic subjects with metabolic disease, in whom 1-h glucose tolerance testing (GTT) revealed a high serum post-glucose load level of ≥155 mg/dl. In these individuals, lower levels of esRAGE and higher levels of RAGE ligand S100A12 were observed vs. control subjects, in whom 1-h post-glucose load level was <155 mg/dl, in parallel with increased pulse wave velocity (PWV) and carotid IMT (41). These data suggested heterogeneity of metabolic dysfunction among subjects within normal limits of glucose tolerance, which might be linked to the RAGE pathway. In a separate study, subjects without a previous history of diabetes were stratified into three groups: controls, pre-diabetes, and new-onset T2D. The prediabetic subjects displayed lower levels of esRAGE and higher levels of S100A12 compared to controls; in the subjects with lower esRAGE, peripheral blood mononuclear cells (PBMCs) demonstrated lower levels of the esRAGE splice variant, suggesting that the lower systemic levels of esRAGE could be accounted for, at least in part, by lower transcription of this splice variant (42). Statistical analyses revealed that age, glycosylated hemoglobin and esRAGE were the major determinants of IMT and levels of S100A12 and blood pressure (systolic) were the main determinants of PWV (42).

In a 3-year longitudinal study of 1,002 subjects with CVD, 933 underwent testing for sRAGE levels, which were then segregated by quartiles. After 3 years follow-up, 16% of the subjects demonstrated a new CVD event (MI, stroke, and CVD death). The patients with the highest quartile of sRAGE displayed the highest incidence of recurrent CVD events, even after correction for confounders for CVD (43).

Collectively, these recent studies add to a large body of reports on the relationship between sRAGEs and diabetes and CVD and suggest the following insights: (1) High levels of sRAGEs may be protective, at least in early stages of disease or, perhaps, in periods of active exacerbation of acute CVD events; and (2) even after the discernment of early metabolic vulnerability subsets in subjects without diagnosed diabetes, the levels of sRAGEs may align with markers of CVD risk. These considerations underscore that long-term prospective studies in subjects without and with varying degrees of metabolic dysfunction are required to fully test if the levels of sRAGEs, including both sRAGE and esRAGE, correlate with CVD predilection, first events and recurrent events.

#### Studies in Animal Models

Early *in vivo* studies in animal models of diabetes and atherosclerosis were performed in mice devoid of *Apoe* and rendered T1D-like with streptozotocin; these mice developed accelerated atherosclerosis in the hyperglycemic state (44). Daily treatment with recombinant sRAGE (by intraperitoneal injection) resulted in a reduction in the development of accelerated atherosclerosis in diabetic mice devoid of *Apoe*, without effects on levels of glucose or lipids. In a distinct study, treatment of diabetic mice devoid of *Apoe* with established atherosclerosis with sRAGE resulted in halting the progress of diabetic atherosclerosis (45). In mice devoid of *Apoe* or *Ldlr*, and in transgenic mice expressing cytoplasmic domain-deleted RAGE [in endothelial cells (ECs)] or in mice with global genetic deletion of *Ager*, significant attenuation in atherosclerosis, irrespective of the diabetic state, but particularly in animals with hyperglycemia, was observed (46–48). Studies using transgenic mice in which the cytoplasmic domain of RAGE was deleted in ECs revealed prominent roles for EC RAGE in endothelial function and signal transduction. RAGE ligands specifically upregulated inflammatory markers, such as Vascular Cell Adhesion Molecule 1 (VCAM1) in ECs from wild-type aorta, but not in ECs lacking the RAGE cytoplasmic domain (46). Bu and colleagues analyzed the transcriptome of the aortas of mice devoid of *Apoe* with or without simultaneous deletion of *Ager* in the T1D state. A significant RAGE-dependent modulation of the ROCK1 branch of the TGF-β signaling pathway in SMCs was uncovered in these analyses, suggesting that SMC RAGE contributed importantly to diabetic atherosclerosis in mice through ROCK1 signaling (47).

Key roles for myeloid *Ager* in diabetic atherosclerosis were also uncovered through bone marrow transplantation studies (49). In macrophages, RAGE ligand-RAGE interaction significantly attenuated cholesterol efflux to APOA1 and HDL and downregulated the cholesterol transporters *Abca1* and *Abcg1*, at least in part through PPAR-γ-dependent regulation of these transporters in both murine bone marrow derived macrophages (BMDMs) and human THP1 cells (50). Beyond genes regulating cholesterol efflux, significant attenuation of vascular inflammation was observed upon deletion of *Ager* in diabetic atherosclerotic mice.

### Vascular Calcification

Diabetes is associated with significant acceleration of vascular calcification, due at least in part to the pathogenic effects of hyperglycemia and oxidative stress (51–54).

#### Studies in Human Subjects

The ligand-RAGE axis has been explored in vascular calcification in human subjects. In 199 patients on hemodialysis in whom vascular calcium scores were obtained (49.2% of the subjects had diabetes), circulating levels of sRAGE were negatively associated with calcium score independent of the level of S100A12 and inflammatory markers (55). In non-diabetic subjects undergoing hemodialysis, levels of esRAGE were significantly lower than those of control subjects and correlated negatively with the degree of aortic calcification (56). In SMCs isolated from the saphenous veins of patients undergoing coronary artery bypass grafting (CABG), exposure to high levels of glucose resulted in NADPH oxidase- and Protein Kinase C-dependent translocation of HMGB1 to the nucleus, which increased calcification through an NF-κB-dependent regulation of bone morphogenetic protein 2 (BMP2) (57). Consistent with roles for RAGE ligands in these processes, exposure of vascular SMCs to AGEs increased calcification, at least in part through activation of p38 mitogen-activated protein kinase (MAPK) (58, 59). Collectively, these considerations suggest that RAGE signaling contributes to vascular calcification in diabetic and non-diabetic settings, presumably on account of the generation of RAGE ligands such as AGEs and other pro-inflammatory/pro-oxidative ligands in conditions such as advanced renal disease.

#### Studies in Animal Models

Studies in mouse models using various means to facilitate calcification underscored roles for RAGE in these processes *in vivo*. Mice devoid of *Apoe* or mice devoid of *Apoe* and *Ager* were subjected to either chronic kidney disease (CKD) or sham surgery and subsets of these animals were fed a high phosphate diet. After 12 weeks of CKD, RAGE ligands AGEs, and S100/calgranulins were increased in the serum of the *Apoe* null mice with a significant increase in *Ager* mRNA in the CKD vessels vs. controls. Vascular calcification was increased in the CKD *Apoe* null mice, in parallel with increased expression of *Runx*, which was lower in mice devoid of *Ager* (60). *In vitro*, stimulation of SMCs with RAGE ligand S100A12 stimulated mineralization and osteoblast transformation, which was inhibited by *Ager* deletion in these cells (60). In other studies, direct mediating roles for S100/calgranulins in the pathogenesis of vascular calcification were illustrated by studies in which transgenic mice overexpressing RAGE ligand S100A12 subjected to CKD demonstrated increased vascular calcification through NADPH oxidase-dependent mechanisms (61).

In valvular calcification (mitral valve and aortic valve) associated with CKD, an upregulation of FGF23 in the heart and vascular tissues was observed selectively in S100 transgenic mice with CKD but not in CKD wild-type or CKD *Ager* null S100 transgenic mice, thereby implicating S100/RAGE in upregulation of FGF23 and pro-inflammatory factors contributing to vascular calcification (62).

In summary, the accumulation of RAGE ligands in both diabetes and non-diabetes states of CKD exacerbates vascular calcification, at least in part through RAGE. The apparently potent mediating influence of the S100/calgranulins, demonstrated directly through the study of S100-transgenic mice, underscores the multi-ligand contributions of the RAGE pathway to CVD and calcification.

### Peripheral Arterial Disease

Peripheral arterial disease (PAD) is increased in patients with T2D and contributes to amputations and substantial morbidity and mortality in affected subjects (63, 64). Unlike cardio- and cerebrovascular disease, PAD is not fully explained by traditional risk factors, perhaps on account of the fact that endothelial, neuropathic and immune/infection-related perturbations also contribute importantly to this disorder and the frequent accompaniment of impaired wound healing (65).

#### Studies in Human Subjects

Accumulating evidence links the ligand-RAGE pathway to the pathogenesis of PAD (66). RAGE ligands S100A12 and carboxymethyllysine (CML)-AGE are elevated in the circulation of subjects with PAD vs. control subjects (67) and, interestingly, in the infrainguinal vein tissue used for vascular grafting in this disorder, the proportion of the tissue stained for AGE, CML, RAGE, and S100A12 was similar in patients with and without diabetes (67), suggesting that non-glucose-related factors also led to the recruitment of the RAGE pathway in PAD. Examination of levels of sRAGE in patients with CAD and/or PAD revealed that the lowest overall levels were observed in patients with both disorders (68) vs. either disorder alone, suggesting that the factors mediating disease in both areas differed, at least in part.

In a population-based cohort study, levels of S100, RAGE ligands, and esRAGE (collectively referred to as RAGE score) were examined in 106 subjects with PAD with and without amputation. The authors reported that higher levels of plasma S100A12 and the overall RAGE score were associated with shorter amputation-free survival in T2D patients, suggesting that the RAGE pathway contributed to the severity of PAD (65).

#### Studies in Animal Models

These concepts have been tested in animal models of PAD, using unilateral hind limb ischemia as a means to introduce ischemic injury to the peripheral vascular system. In those studies, key endpoints include angiogenesis responses and detection of blood flow by Laser Doppler Imaging techniques. In a mouse model of hind limb ischemia, using multimodal imaging with molecularly targeted nanoparticles, a significant increase in RAGE expression accompanied hind limb ischemia vs. the sham limb (69). Others showed that RAGE imaging (using an anti-RAGE antibody fragment) was enhanced in diabetic vs. non-diabetic hind limb ischemia (70).

Global deletion of *Ager* and administration of anti-RAGE antibodies have been shown to improve angiogenesis and blood flow recovery in diabetic mice; in mice globally devoid of *Ager*, the beneficial effects were observed in hind limb ischemia induced in both diabetic and non-diabetic animals (71–73).

The model of hind limb ischemia underscored interesting distinctions vis-à-vis RAGE signaling in atherosclerosis vs. the peripheral vascular system. Whereas, it was shown that immune cell content (macrophages and T cells) was significantly reduced in the atherosclerotic lesions of diabetic *Apoe* null mice devoid of *Ager* vs. diabetic *Apoe* null mice expressing *Ager* (47), macrophage content in the peak period of immune cell infiltration into the ischemic hind limb was significantly higher in *Ager* null vs. wild-type animals, in both the diabetic and non-diabetic states (71). In parallel, the ischemic hind limb muscle tissue of *Ager* null mice displayed significantly higher mRNA transcripts for *Ccl2* and *Egr1*, genes involved in inflammatory cell recruitment and pro-inflammatory mechanisms (71).

Despite the apparent differences in RAGE impact on immune cell content in atherosclerosis vs. hind limb ischemia, in both settings, deletion of *Ager* (and administration of anti-RAGE antibodies in T1D mice subjected to hind limb ischemia) resulted in reduced vascular disease. These findings underscore the complexity and plasticity of RAGE signaling in immune cells in distinct vascular depots and in *in vivo* conditions and suggest that niche-specific cues regulate RAGE ligands and RAGE responses in distinct forms of vascular injury.

### Atrial Fibrillation

Cardiac rhythm abnormalities such as atrial fibrillation (AF) accompany disorders of the cardiovasculature. It has been suggested that the incidence of AF is higher in diabetic patients, especially those with longer disease duration or poor glycemic control and that RAGE ligand AGEs, through their ability to increase stiffness, oxidative stress, and fibrosis contribute to this phenomenon (74). Markers of the ligand-RAGE pathway were investigated in human subjects with AF. When comparing 38 patients with AF vs. 59 in normal sinus rhythm, it was shown that levels of fluorescent AGEs and sRAGE were higher in the AF vs. control subjects, especially in non-diabetic patients (75). The markers of AGEs and sRAGE correlated with left atrial dimensions in that study (75). In a distinct study, levels of plasma sRAGE and esRAGE were found to be higher in Caucasian patients with persistent AF vs. paroxysmal AF (76). In another study examining the effects of therapeutic intervention, higher plasma levels of sRAGE were independently associated with reduced rate of recurrence of AF after catheter ablation in diabetic patients (77).

In contrast, in the Atherosclerosis Risk in Communities (ARIC) study, 1068 participants were studied who had baseline sRAGE values determined at the time of study entry. Multiple measures of inflammation were also obtained in these subjects. Compared to the highest quartile of sRAGE, the lowest quartile of sRAGE was associated with the higher baseline levels of inflammatory markers (hsCRP, white blood cell count and fibrinogen). However, when viewed prospectively (6-year change in inflammatory markers), there was no association with sRAGE. Moreover, no significant associations of sRAGE levels were noted with the risk for AF (78).

Collectively, these studies suggest that at least in certain populations and certain conditions, levels of sRAGE may be biomarkers for AF. More work is needed to definitively test these concepts.

### Thrombotic Disorders

There is published evidence suggesting links between diabetic complications and thrombosis and platelet pathobiology. For example, examination of mean platelet volume (MPV) revealed that this measure was higher in human T2D subjects with uncontrolled hyperglycemia and a statistically significant association between MPV and albuminuria was also documented (79). High levels of glucose were linked to increased NETosis (neutrophil extracellular traps) and it is suggested that NETosis is associated with T2D (80). In a meta-analysis of the adverse outcomes occurring after Percutaneous Coronary Intervention (PCI) involving >139,000 subjects, it was identified that short-term stent thrombosis, but not long-term stent thrombosis, was significantly higher in subjects with diabetes vs. the non-diabetic control subjects (81). Others studied platelets and their characteristics in diabetes and reported that in T2D, significantly higher platelet activation and markers of hypercoagulation were observed, with increased platelet expression of GP11b/IIIa receptors (82). However, the MEGA study (Multiple Environmental and Genetic Assessment) failed to identify relationships between self-reported diabetes, fasting levels of blood glucose, and venous thrombosis (83).

Emerging evidence links RAGE and at its ligands, AGEs, S100/calgranulins, and HMGB1, to thrombosis and thrombotic disorders. For example, increased expression of HMGB1 has been observed in a number of thrombosis-related diseases such as CAD, stroke, PAD, disseminated intravascular coagulation (DIC), and venous thrombosis (84). The biology of HMGB1 is complex in that, in addition to binding to RAGE, HMGB1 is also a ligand for some of the toll-like receptors (TLRs) (85). Recent studies have probed potential mechanistic links between RAGE and thrombotic disorders.

#### Studies in Human Subjects

In the prothrombotic disorder known as anti-phospholipid syndrome (APS), it has been established that one of the main targets of the anti-phospholipid antibody is β_2_ glycoprotein 1 (or anti-β_2_-GP1). When platelets and monocytes obtained from healthy human subject donors were incubated with anti-β_2_-GP1, upregulation of RAGE was noted as well as altered cellular location of HMGB1 (86). In serum studies, levels of sRAGE and HMGB1 were significantly higher in patients with APS vs. controls and there was a direct correlation between the levels of HMGB1 and disease duration (86).

Other studies illustrated that HMGB1 binds to platelets and that platelet activation resulted in upregulation of RAGE expression and that HMGB1 was highly expressed in platelet-rich human coronary artery thrombi (87). In ECs obtained from human saphenous veins, incubation with AGEs increased neutrophil adhesion and generation of reactive oxygen species (ROS) and treatment with simvastatin reduced these prothrombotic stimuli and reduced RAGE expression (88). ECs from diabetic patients treated with simvastatin resulted in reduced expression of RAGE, neutrophil adhesion and ROS (88). However, other studies tested if the levels of platelet HMGB1 were associated with outcomes in symptomatic CAD. The authors reported that there were no differences in platelet expression of HMGB1 when comparing patients with stable CAD, unstable CAD, non-ST segment elevation myocardial infarction (NSTEMI), or ST segment elevation myocardial infarction (STEMI) (89). Further, there were no correlations between left ventricular ejection fraction (LVEF) amongst the subjects that also suffered MI.

HMGB1 derived from platelets also affected monocyte behavior; it was shown that HMGB1 trigged monocyte migration via RAGE and suppressed monocyte apoptosis through a TLR4-dependent activation of the MAPK pathway in these cells (90). Hence, platelet HMGB1-RAGE interactions might impact on distinct cell types, which collectively contribute to the increased risk and severity of CVD.

#### Studies in Animal Models

In animal models, neutrophil-derived S100A8/A9, through liver production of IL6, promoted production of thrombopoietin, which resulted in reticulated thrombocytosis, was found to be increased in diabetic animals, but reduced by lowering of blood glucose using dapagliflozin or by blocking the binding of S100A8/A9 to RAGE using paquinimod, in mice (91). These authors correlated their findings in human diabetic subjects in whom reticulated thrombocytosis correlated both with the levels of glycosylated hemoglobin and S100A8/A9 (91).

Other studies in mice illustrated that disulfide HMGB1 facilitated monocyte recruitment and, through RAGE, stimulated the formation of the prothrombotic NETs; this process then exposed additional HMGB1 on their extracellular DNA strands to propagate the prothrombotic effects of HMGB1 and NETs (92).

Collectively, these studies link RAGE and its ligands to platelet perturbation and to upregulation of prothrombotic mechanisms, which are both associated with and independent of diabetes, but linked to the complications of diabetes in the cardiovascular system.

### Myocardial Infarction

MI is a critical complication of diabetes, which occurs to accelerated rates and degrees in patients with diabetes. Studies in animal models have forged insights into roles for the ligand-RAGE axis in the pathogenesis of diabetic CVD and MI. Emerging insights from human subjects now link this axis to MI as well.

#### Studies in Human Subjects

Studies in human subjects with MI and related CVD disorders have been probed for the ligand-RAGE axis. In subjects from Japan with T2D, baseline clinical, and biochemical data were examined and prospectively evaluated for the association between those parameters and CVD events over a mean follow-up period of 5.6 years with 25 new CVD events reported during that time. In a tertile analysis, the risk for CVD events rose with increasing levels of sRAGE; a multivariate Cox proportional hazards regression analysis showed that even after correction for typical coronary risk factors, serum sRAGE levels remained independently associated with CVD (93). In a registry of patients enrolled during 2009–2011 with acute MI, the mean values of fluorescent AGEs and CRP did not differ between diabetic and non-diabetic subjects with MI; however, a direct association between AGE levels and CRP was observed only in diabetic, but not non-diabetic patients (94). In patients who had received statins before their MI, however, this relationship disappeared (94), suggesting that statin therapies might mitigate the impact of proinflammatory stimuli. In a distinct study in patients with acute coronary syndrome (ACS), plasma levels of sRAGE were significantly lower in subjects with ACS vs. stable angina pectoris. These authors showed, however, that in the subjects with ACS, the levels of sRAGE did not correlate with the number of affected vessels (95). These considerations suggest that sRAGE might be a biomarker of plaque destabilization but not necessarily the extent of plaque burden in human CAD.

Others examined a group of subjects with T2D undergoing sirolimus-stent PCI. The primary endpoint for the study was the MACCE, or major adverse cardio-cerebral events, which included the following: cardiac death, non-fatal MI or non-fatal stroke during a 2-year period of follow-up. The secondary endpoint of the study was the need for clinically-driven repeat revascularization during the 2-year period. The authors monitored levels of circulating glycated albumin and esRAGE and found that both glycated albumin and esRAGE predicted long-term clinical outcomes; specifically, elevated serum glycated albumin and reduced esRAGE were associated with poor clinical outcomes in this patient group (96). In a distinct study in patients with T2D undergoing drug-eluting stent implantation, the relationship between plasma levels of sRAGE and in-stent restenosis were probed and measured at the time of the stent implantation. The authors reported that plasma levels of sRAGE were significantly higher in the T2D patients with in-stent restenosis vs. control T2D subjects; interestingly, the levels of glycosylated hemoglobin, CRP, and IGF-1 (insulin-like growth factor 1) did not differ between the groups (97). When the authors performed multivariate regression analysis, they found that plasma levels of sRAGE and mean stent diameter <2.0 mm significantly predicted in-stent restenosis (97).

A critical complication of acute MI is the development of cardiogenic shock. As it was shown that higher levels of monocyte RAGE and lower levels of plasma sRAGE were linked to higher mortality in cardiogenic shock, the effects on levels of MMP9 and Tissue Inhibitors of Metalloproteinases (TIMPs) were tested, as MMPs have been shown to contribute to the production of sRAGE through the cleavage of its extracellular domains. MMP9 activity was found to be increased in acute MI survivors but reduced in subjects with acute MI who developed cardiogenic shock (98). Further, MMP9 activity was found to correlate inversely with RAGE expression on monocytes. Collectively, the above considerations suggest that sRAGE might serve as a biomarker in acute MI with respect to prognosis and that maintenance of effective MMP9 activity may serve to stabilize sRAGE production in MI complicated by cardiogenic shock.

With respect to RAGE ligand HMGB1, plasma levels of this factor were shown to be related to infarct size and to residual left ventricular function after MI (99).

#### Studies in Animal Models

Studies in the isolated perfused heart and in *in vivo* MI triggered by occlusion/reperfusion of the left anterior coronary artery in rats and/or mice have shown that blockade of RAGE, using either sRAGE or in genetically modified mice, that is *Ager* null mice or transgenic mice expressing cytoplasmic domain-deleted RAGE (in macrophages or ECs) is protective against ischemia/reperfusion (I/R) injury in diabetic and non-diabetic animals (100–102). In those studies, reduced infarct size, reduced myocardial necrosis, and increased cardiac function and ATP recovery accompanied blockade of the RAGE axis. In cultured cardiomyocytes, induction of hypoxia/reoxygenation (H/R) stimulated RAGE-dependent activation of JNK MAP kinase and dephosphorylation of GSK-3β, which was prevented in cells devoid of *Ager* or upon treatment with sRAGE in wild-type cardiomyocytes (103).

Others employed a rat model of MI and cultured cardiomyocytes to study the S100/calgranulin-RAGE interactions and showed that S100B via RAGE may contribute to cardiomyocyte apoptosis via activation of ERK1/2 MAPK and p53 signaling (104). In a distinct study in T1D mice, coronary artery ligation was performed in wild-type and S100B-deleted mice. Diabetes and MI induction each alone induced expression of S100B and RAGE in the heart; but in the post-MI myocardium, only in diabetic mice, the expression of S100B was attenuated. In the diabetic S100B-deleted mice post-MI, increased dilation of the left ventricle was noted compared to diabetic wild-type mice, in parallel with increased impairment of cardiac function, expression of GLUT4 and systemic levels of AGE (105). Collectively, those studies suggested that S100B expression may beneficially modulate cardiac metabolism post-MI in diabetes. Distinct studies also implicated ligand HMGB1 in experimental cardiac MI, at least in part through RAGE, in both diabetic and non-diabetic animals (106). Hence, identification of the timing of the actions of the specific RAGE ligands in the chronic vs. acute setting appears to be essential in order to discern the optimal conditions for RAGE antagonism in MI.

Finally, studies have begun to examine the potential roles of the formin, DIAPH1, the cytoplasmic domain binding partner of RAGE, in myocardial I/R injury. After induction of I/R in wild-type mice, DIAPH1 expression was upregulated; in cultured H9C2 and AC16 cardiomyocytes, H/R also upregulated expression of DIAPH1 (32). Consistent with mediating roles for DIAPH1 in myocardial injury, global deletion of *Diaph1* reduced infarct size and preserved cardiac function after experimental MI when compared to the *Diaph1*-expressing control animals. In H9C2 cells, silencing of *Diaph1* in H/R reduced expression of the sodium-calcium exchanger and increased sarcoplasmic calcium ATPase activity (32).

### Fat Depots, RAGE, Obesity, and CVD

Recent work has highlighted roles for distinct fat depots, such as brown adipose tissue, subcutaneous adipose tissue (SAT), visceral (omental/epididymal), perivascular adipose tissue (PVAT), and epicardial adipose tissue (EAT) in cardiometabolic fate in human subjects and animal models. Intriguingly, RAGE contributes to metabolic perturbation via its expression in multiple fat depots.

#### Studies in Human Subjects

In human omental adipose tissue, obesity was associated with increased accumulation of CML-AGE RAGE ligand and RAGE expression compared to lean individuals; interestingly, it was reported that CML-AGE levels were reduced in the circulation of obese subjects, which was proposed to be due to tissue trapping of CML-AGE in the adipose tissue, at least in part on account of higher RAGE expression (107). These decreased levels of circulating CML-AGE were also shown to correlate with insulin resistance (107).

Increasingly, epicardial adipose tissue or EAT has been linked to CVD (108, 109). In 33 human subjects undergoing open-heart surgery, EAT was retrieved for analyses. As RAGE expression rose, increased EAT thickness, reduced expression of GLUT4, adiponectin and glyoxalase1 (GLO1), and elevated levels of HMGB1, TLR4, and MYD88 were observed (110), suggesting that the ligand-RAGE axis may be associated with EAT adiposity and metabolic dysfunction. In a distinct study, SAT and EAT were obtained for RNA-sequencing from 5 T2D patients with CAD and 3 subjects without CAD with or without T2D undergoing cardiac surgery. 592 genes were differentially expressed in diabetic vs. non-diabetic EAT; there were no changes in the transcriptome between diabetic and non-diabetic SAT (110). The diabetic EAT-associated genes were largely linked to inflammation (IL1B and IL6); KEGG pathway analysis placed these differentially-expressed genes in the TNF, NF-κB, and AGE-RAGE pathways (110).

Imaging modalities have also been employed to track epicardial fat volume (EFV), paracardial fat volume (PFV) and perivascular fat (PVAT) in 66 consecutive patients (33 with diabetes) and multivessel CAD included for study. In diabetes, higher EFV was observed; at the transcript level, patients with diabetes displayed significantly higher RAGE expression in EAT (111).

#### Studies in Animal Models

Similar observations have been made in animal models; even prior to the onset of high fat diet-induced obesity and the development of insulin resistance in wild-type mice, RAGE ligands are upregulated in the metabolic organs (112). On account of these observations, the potential roles of RAGE in diet-induced obesity were studied by feeding mice a high-fat diet (60% kcal from fat).

In mice globally devoid of *Ager*, mice fed a 60% high-fat diet were significantly protected from the gain in body mass that accompanied the feeding of this diet in wild-type mice (112). In parallel, the *Ager* null mice were protected from insulin resistance that accompanied obesity in the wild-type animals; this was determined both through insulin tolerance tests (intraperitoneal injections of glucose) and through the hyperinsulinemic euglycemic clamp (112). Indirect calorimetry studies revealed that food intake did not differ between the two genotypes of mice fed the high-fat diet, but energy expenditure was significantly higher in the mice devoid of *Ager* vs. the control animals. Pharmacological blockade of RAGE, using sRAGE, in wild-type mice, significantly suppressed weight gain when compared to vehicle upon introduction of sRAGE either immediately at the time of the high-fat diet feeding or 3 weeks after the initiation of the high-fat diet (112). These results led to the direct testing of roles for RAGE in regulation of energy expenditure, focusing on the adipocyte.

*Ager* floxed mice were bred into the *Adiponectin (Adipoq)* Cre recombinase background, which resulted in deletion of *Ager* in both brown and white adipose tissue adipocytes. These mice were significantly protected from high-fat diet-induced obesity and from cold-induced loss of body temperature when compared to *Ager* floxed control mice, in which RAGE was expressed in the adipocytes (113). The underlying mechanisms were traced to RAGE ligand-RAGE-dependent suppression of lipolysis and thermogenic programs (such as expression of *Ucp1*) in these settings, through reduced phosphorylation of p38 MAP kinase and hormone sensitive lipase (HSL) (113). On account of the fact that the *Adipoq* Cre recombinase mice could not discern mechanistic roles for RAGE in white vs. brown adipose tissue, adipose tissue transplantation of either brown or subcutaneous white adipose tissue from these mice, or their controls, were introduced into wild-type mice C57BL/6J recipients. Transplantation of adipocyte *Ager*-deficient brown or subcutaneous white adipose tissue protected the recipient mice from obesity induced by high-fat feeding via upregulation of thermogenic programs. Interestingly, in both cases, the native brown or white adipose tissue of the recipients of the *Ager*-deleted adipocytes (white or brown) displayed increased expression of UCP1 protein by immunostaining, suggesting that the transplanted tissue conferred its beneficial effects, at least in part through paracrine mechanisms that directly and beneficially affected the native brown and subcutaneous white fat depots (113).

Collectively, these results identified a natural function for RAGE in energy conservation mechanisms and suggested that the cardiometabolic effects of the RAGE signaling pathway are active even before the development of T2D.

Increasingly, studies in human subjects demonstrate genetic associations of *AGER* and *DIAPH1* SNPs to disease. In the sections to follow, we detail the findings on *AGER* and *DIAPH1* SNPs in cardiometabolic disease.

## RAGE and DIAPH1 and SNPs—Deepening the Connections to Human Subjects

### *AGER* SNPs

Multiple SNPs of the *AGER* gene have been described; among the most common include the following: rs2070600, rs1800624, rs1800625, rs184003, and a 63 bp deletion (114, 115). The rs2070600 represents the nucleotide change 244G>A and at the amino acid level, Gly82Ser (114). This *AGER* SNP was of particular interest on account of the fact that the Gly82Ser is within the V-type Ig domain, that is, the extracellular domain encompassing much of the ligand binding capacity (116). Structurally, the Gly82Ser SNP promotes N-linked glycosylation of Asn81, which is important for RAGE ligand binding (117). Indeed, *in vitro*, cultured cells bearing the RAGE 82S allele displayed enhanced binding affinities for RAGE ligands in the S100/calgranulin family, and upon RAGE ligand stimulation, exaggerated expression of cytokines and MMPs was observed in G82S- vs. G82G-transfected cells, suggestive of an amplified inflammatory response (116). With respect to human subjects and inflammatory disease, a case-control study revealed that there was an increased prevalence of the 82S allele in patients with rheumatoid arthritis (RA) compared with control subjects (116).

Other SNPs, specifically, rs1800624 (-388T>A), rs1800625 (-442T>C), and rs1051993 (-1435G>T); and a 63 base pair deletion (-421_-359) reflect promoter variants and rs184003 (822+49G>T) affects intron 7–8 (114).

Review of the literature suggests that the links of *AGER* SNPs to cardiovascular disease (CVD) may be dependent on ethnicity (114). For example, extensive studies in the Chinese Han population suggested that the rs2070600 SNP was significantly associated with increased risk of all-cause mortality and acute myocardial infarction (MI) (118). Ma and colleagues performed a meta-analysis of 16 eligible studies reporting on rs2070600 SNP and CVD; they reported an association between this SNP and coronary artery disease (CAD) and ischemic stroke (IS) in the Chinese population, but not in non-Chinese populations (119). Until studies examining larger groups of subjects are examined to fully test this *AGER* SNP and potential relationships to CVD, studies of these specific populations in China may, therefore, shed light on mechanisms of RAGE-dependent predilections to CVD.

### *DIAPH1* SNPs

Compared to *AGER*, at least to date, less is reported with respect to *DIAPH1* SNPs and human disease. However, a report linked a *DIAPH1* SNP to a blood-related disorder. The R1213^*^ variant of *DIAPH1* was associated with sensorineural hearing loss and a disorder of platelets, called macrothrombocytopenia (MTP), in which cytoskeletal abnormalities in megakaryocytes (platelet precursors) and platelets were described (120). This mutation, which affects the autoregulatory domain of DIAPH1, results in constitutive activation of DIAPH1 (120).

Recently, *DIAPH1* SNPs were linked to stroke. One of the *DIAPH1* SNPs, Rs7703688T>C was significantly associated with increased risk of ischemic stroke, *p* = 4.139 × 10^12^, which was further validated in an additional group (121). Further, in a small artery occlusion (SAO) subtype of stroke, *DIAPH1* expression in affected tissue displayed trends to increased levels in subjects with the rs25019 genotype, p_trend_ =0.048 (121). These findings add to the connections between DIAPH1 and human subjects in the context of CVD.

Collectively, these data, from animal models and human subjects suggest roles for RAGE/DIAPH1 in cardiometabolic disease. Hence, efforts to target RAGE pharmacologically might provide substantial benefit in obesity, metabolic dysfunction, diabetes, and diabetic complications.

## Targeting RAGE Through Blockade of RAGE-DIAPH1 Interaction

As indicated earlier in this review, the extracellular domains of RAGE (V, C1, and C2-type Ig domains) bind the diverse classes of ligands in distinct sites; although the V-type Ig domain is the chief site for ligand interaction, the C1- and C2-type Ig domains are reported to bind ligands as well (11–14). Hence, it was perhaps not surprising that the small molecule known as Azeliragon failed to show benefit in a Phase III clinical trial in Alzheimer Disease (AD) for subjects with mild cognitive impairment (MCI) when compared with placebo-treated patients (122). In AD, beyond RAGE ligand Aß, it is known that multiple classes of pro-inflammatory and pro-oxidative RAGE ligands are enriched in the AD brain (123, 124).

**Figure 1:**
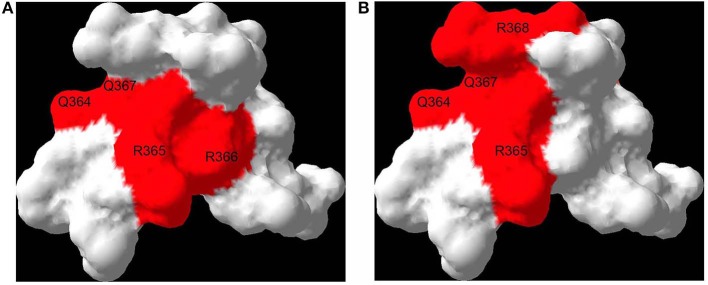
RAGE binds DIAPH1: effect of a small molecule antagonist. Both DIAPH1 and a small molecule RAGE antagonist bind to the proximal sites on the RAGE cytoplasmic domain, suggesting a mechanism of RAGE inhibition. Interaction surfaces of DIAPH1-RAGE [see (23)] **(A)** and small molecule RAGE antagonist-RAGE [see (124)] **(B)** are mapped onto a solution structure of RAGE cytoplasmic domain (PDB code 2lmb). Affected residues are labeled. The residues are numbered based on the full length RAGE.

In this context, the demonstration of the binding of the RAGE cytoplasmic domain to DIAPH1 in a discrete manner, with a binding pocket <200 Å, paved the way for the development of small molecule antagonists for this interaction as a means to block RAGE signaling (23). Accordingly, a 59,000 small molecule library was screened to identify inhibitors of the interaction of the RAGE cytoplasmic domain with DIAPH1; 11 such molecules were reported on the basis of their ability to bind to the RAGE cytoplasmic domain and block DIAPH1 binding (Figure 1); the ability to block RAGE ligand-mediated signaling stimulated by multiple different classes of ligands; the ability to block RAGE ligand, but not non-RAGE ligand-mediated cellular migration in SMCs; the ability to reduce I/R injury in the isolated perfused heart model; and the ability to block the proinflammatory actions of RAGE ligands (CML-AGE) injected into wild-type mice (125). Further development and refinement of the key scaffolds identified from that work is underway at this time. If successful, such efforts may result in the development of a novel class of RAGE antagonists.

## Perspectives and Challenges

Despite multiple advances for therapeutic interventions in CVD, gaps in therapies remain for subjects with diabetes, in whom traditional treatments do not afford the same degree of protection as they do in non-diabetic subjects. In this context, the identification of diabetes-specific disease-modifying pathways is essential to fill the chasm in therapeutic opportunities for patients with diabetes. Further, the identification of the optimal timing for intervention with disease-modifying agents is essential in order to maximize potential benefits, even if preventive therapies are, ultimately, deemed more realistic, as they are for hyperlipidemia and hypertensive disorders, for example.

Hence, in diabetes, it is logical that targeting the consequences of the defining feature of the disorder, that is, hyperglycemia, may yield benefit. The acute and long-term effects of high glucose, manifested in part by the formation of AGEs, is one such target. As AGEs form and accumulate both endogenously and through dietary ingestion, and as AGEs represent a highly heterogeneous class of structures, the direct targeting of AGEs may be challenging. For this reason, antagonizing the cellular effects of AGEs may be more comprehensive and feasible. Complicating this notion, however, is the finding that the chief receptor for AGEs, RAGE, is a multi-ligand receptor whose ligands display significant promiscuity in their binding modes to the extracellular domains of RAGE. Thus, inhibiting the interaction of the RAGE cytoplasmic domain with DIAPH1 may reflect a superior approach by curating the effects of diverse ligands signaling through RAGE/DIAPH1. Indeed, significant advances in the development of protein-protein interaction inhibitors (PPI) in other settings bolster promise for this proposed approach (126, 127).

**Figure 2:**
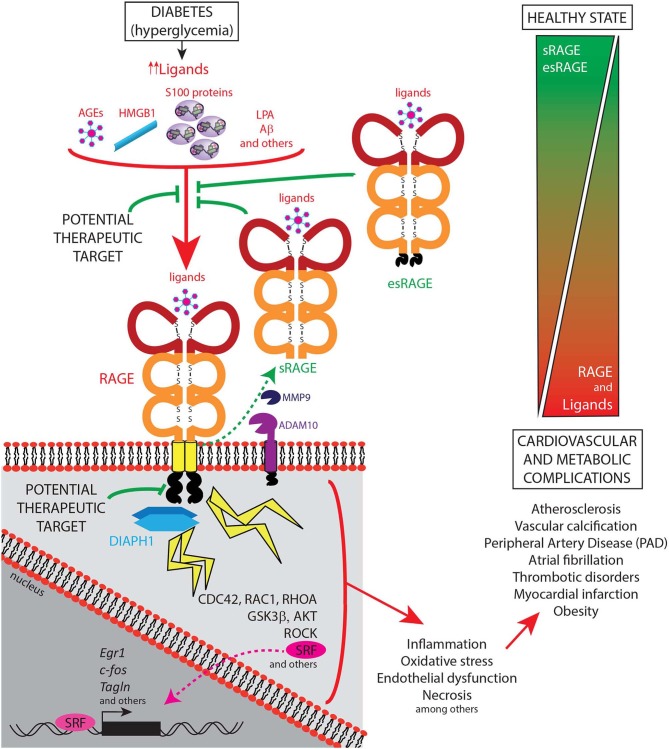
Schematic representation of the ligand-RAGE-DIAPH1 axis and its role in diabetic cardiometabolic complications. The receptor for advanced glycation end products (RAGE; gene name is *AGER*) mainly acts through its known ligands, such as AGEs, HMGB1, S100 family of proteins, LPA, and Aβ, which bind the RAGE extracellular domains. The cytoplasmic domain of RAGE interacts with its cytoplasmic effector protein, Diaphanous1 (DIAPH1), thereby activating multiple downstream regulators and stress responses, as illustrated in the figure. Cellular stressors such as inflammation, oxidative stress, endothelial dysfunction, and necrosis amongst others, are well-known induce cardiovascular and metabolic complications such as atherosclerosis, vascular calcification, peripheral artery disease, atrial fibrillation, thrombotic disorders, myocardial infarction, and obesity. In contrast, soluble forms of RAGE, including sRAGE, that results from cell surface-cleavage of the full-length receptor by Matrix Metallopeptidase-9 (MMP9) and A Disintegrin And Metalloproteinase Domain-Containing Protein-10 (ADAM10), and esRAGE, a product of a splice variant of *AGER*, have been demonstrated to show a protective role in cardiometabolic complications, at least in part, by preventing the RAGE ligands from binding to the cell surface receptor, and, therefore, reducing the RAGE-DIAPH1 signaling activation. Hence, currently identified potential therapeutic targets include blocking the binding of the ligands to the receptor and by interruption of the RAGE-DIAPH1 interaction.

Of note, studies in human subjects underscore that the measurement of the levels of sRAGE and/or esRAGE might provide a biomarker to track the activity of the RAGE pathway (Figure 2). In chronic disease without exacerbation, the levels of sRAGEs appear, in general, to be lower than those in control subjects. However, it is plausible that in exacerbations of chronic disease, such as acute cardiac events, levels of sRAGEs might rise, perhaps in response to increased MMP and ADAM activities, although these relationships remain to be definitively discerned. It is clear, however, that studies testing both sRAGE and esRAGE, in a serial manner, prior to and at the time of and after acute cardiac events will be required to fully discern the reasons for the apparent variability of these markers.

Beyond immediate benefits for antagonizing RAGE/DIAPH1 in cardiometabolic disease in the periphery, it is plausible that targeting the diverse signaling platform through DIAPH1 and stimulated by RAGE ligands may be broadly beneficial, including in the central nervous system. Beyond AGEs and proinflammatory ligands, RAGE is also a receptor for oligomeric forms of Aß, which is strongly implicated in the pathogenesis of AD (128). The relatively recent designation of VCID, or “vascular contributions to cognitive impairment and dementia” culls the collective impact of aging, age- and lifestyle-related disorders (such as hyperlipidemia, hypertension, elevated fasting blood glucose, and obesity) and AD into a schema that suggests that these disorders may complicate and exacerbate each other, with a final common manifestation of “dementia” (129–131). Potentially biomarked by “white matter hyperintensities” or WMHs by imaging techniques, VCID may represent the product of the sum total of vascular/cognitive risks in individuals. It was recently shown that DIAPH1 is upregulated in the brains of AD vs. age-matched control human subjects; that its expression co-localizes with that of RAGE; and that the AD-specific upregulation of DIAPH1 was localized to microglia (132), the endogenous/resident yolk sac-derived immune/inflammatory cells of the brain. In this context, the demonstration of roles for RAGE ligands/RAGE in pathological aging, vascular and cognitive disturbances may suggest broader benefits for RAGE/DIAPH1 antagonism in aging and cardiometabolic disease.

In summary, the identification of fundamental roles for RAGE in energy conservation mechanisms, which go awry in nutrient excess, thereby contributing to the development of obesity in high-fat feeding in mice; and in the propagation of chronic tissue-damaging pro-inflammatory mechanisms, lay the framework for the potential benefits for RAGE antagonism both in the causes and consequences of diabetes and its complications, particularly in CVD. Despite the finding that the highest levels of RAGE expression are in the lung, a plethora of evidence suggests that RAGE plays pathogenic roles in this organ, as RAGE is implicated in such disorders as allergic airway inflammation and asthma, pulmonary fibrosis, lung cancer, chronic obstructive pulmonary disease, acute lunginjury, pneumonia, cystic fibrosis, and bronchopulmonary dysplasia (133). We speculate that therapeutic interruption of RAGE, post-development and in the setting of the mature lung, and with partial antagonism to be achieved by pharmacological means, is very likely to be safely tolerated in the lung and in the overall organism. Further, the observations that deletion of *Ager* is protective in polymicrobial sepsis, massive liver injury and in most forms of infection (134, 135), at least in animal models, suggest that targeting RAGE may, on balance, exert salutary benefits in chronic diseases such as diabetes. These concepts remain to be tested definitively in human subjects with diabetes and cardiometabolic disease and the results of these investigations are eagerly awaited.

## Author Contributions

AMS wrote the first draft of the manuscript and completed all of the editing. LE-G, RL-D, GY, LR, SR, PG, AS, and RR provided critical comments on the manuscript and edited the manuscript.

### Conflict of Interest

AMS, RR, and AS hold submitted and pending patents on intellectual property (small molecule antagonists of RAGE/DIAPH1), which are discussed in this manuscript. The remaining authors declare that the research was conducted in the absence of any commercial or financial relationships that could be construed as a potential conflict of interest.
